# Psychological Consequences of Covid on Health Care Workers and their Coping strategies

**DOI:** 10.1192/j.eurpsy.2023.890

**Published:** 2023-07-19

**Authors:** M. Theodoratou, A. Miari, N. Nikitidis, I. Farmakopoulou

**Affiliations:** 1 Social Sciences, Hellenic Open University, Patras, Greece; 2 Health Sciences, Neapolis University of Pafos, Pafos, Cyprus; 3 Department of Nutrition & Dietetics, University of Thessaly, Karditsa; 4 Education and Social Work Sciences, University of Patras, Patras, Greece

## Abstract

**Introduction:**

Research findings show that during the COVID-19 pandemic, healthcare workers (HCWs) have been subject to increased workload while also exposed to many psychosocial stressors.

**Objectives:**

The aim of this study was to investigate Covid’s impact on healthcare professionals’ mental health and their coping strategies

**Methods:**

The study population consisted of 144 health professionals from health care facilities in Patras. An internet based questionnaire was distributed,which included demographic survey questions and the following three scales: (1) The Psychological Consequences Questionnaire (PCQ) scale, (2) The Kessler Psychological Distress scale (k6) and (3) Toulouse’s scale for coping strategies (E.T.C.).

**Results:**

144 health care workers participated in the survey, who were basically women (72.2%) and nurses (60%) In terms of psychological consequences, participants felt pressured, stressed (3.12), and sad/depressed (2.78). The most frequently used coping strategies were acceptance (3.44), active focus (3.38), cognitive focus (3.31), cognitive control and planning (3.30), emotional control (3.17), social informational support (3.16) and cooperation (3.15). In contrast, the strategies used to a lesser extent are substance addiction (1.91), emotional focus ( 2.13), denial (2.27) and alexithymia (2.49). Generally, positive strategies (3.11) were chosen to a greater extent than negative ones ( 2.38).

**Conclusions:**

It is very important for hospital administrations to design specific psychological support programs and encourage health professionals to participate in them in order to manage their fear, anxiety and stress experienced.

**Disclosure of Interest:**

None Declared

